# Malignant craniopharyngiomas: Institutional experience and literature review

**DOI:** 10.1111/bpa.70068

**Published:** 2026-01-05

**Authors:** Thomas J. Auen, Isabella W. Zhang, Weiwei Zhang, Jordan M. Burr, Matthew L. Carda, James L. Wisecarver, Nicole Shonka, Jesse L. Cox, Sahara J. Cathcart, Allison Cushman‐Vokoun, Jie Chen

**Affiliations:** ^1^ Department of Pathology, Microbiology and Immunology University of Nebraska Medical Center Omaha Nebraska USA; ^2^ Neuroscience Columbia University New York New York USA; ^3^ Department of Pathology Duke University Medical Center Durham North Carolina USA; ^4^ Department of Pathology University of Iowa Hospitals and Clinics Iowa City Iowa USA; ^5^ Department of Internal Medicine University of Nebraska Medical Center Omaha Nebraska USA

**Keywords:** BAP1, CTNNB1, malignant craniopharyngioma, next‐generation sequencing, radiation, TP53

## Abstract

Malignant craniopharyngiomas, de novo or via malignant transformation, are exceedingly rare with a dismal prognosis and unclear treatment standards. Little is known about the factors involved in their pathogenesis. A natural language search, performed in our institutional CoPath system, identified 65 adamantinomatous craniopharyngiomas from 56 patients (25 males, 31 females; median age at initial diagnosis = 22 years). Among those, a unique case of malignant craniopharyngioma was identified in a 36‐year‐old male initially diagnosed with a benign adamantinomatous craniopharyngioma 16 years prior. A literature review identified 44 cases of malignant craniopharyngiomas (current case included) with a median age of 28 years and a median overall survival of 6 months, independent of sex, age, histologic variant, tumor size, or radiation therapy. Eighteen (41%) malignant craniopharyngiomas occurred in patients without a history of radiation, suggesting mechanisms other than radiation contribute to their pathogenesis. Since BRCA1‐Associated Protein 1 (BAP1) and TP53 mutations have recently been reported in a case of malignant craniopharyngioma, we assessed these genes in the current case. Next‐generation sequencing identified variants in BAP1 (c.1850delGinsCA;p.R617fs), TP53 (c.428delT;p.V143fs), and CTNNB1 (c.110C>T;p.S37F). In conclusion, our results demonstrate that malignant craniopharyngioma tends to occur in young adults with a median overall survival of only 6 months. The current case is the second reported to harbor BAP1 and TP53 mutations by sequencing. BAP1 and TP53 mutations may play an important role in the pathogenesis of malignant craniopharyngioma and may offer potential targets for therapeutic intervention.

## Graphical abstract

We report the second case of malignant craniopharyngioma with BAP1 and TP53 mutations. A literature review identified 44 cases of malignant craniopharyngiomas with a median overall survival of 6 months. Eighteen (41%) occurred in patients without any history of radiation, suggesting that mechanisms other than radiation have contributed to their pathogenesis.

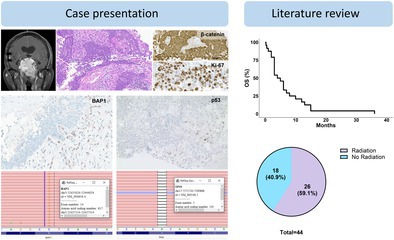

## 1 INTRODUCTION

Adamantinomatous craniopharyngiomas are squamous epithelial tumors of the sellar region derived from mucosal rests of the oral cavity or Rathke's pouch remnants [1, 2, 3, 4, 5, 6]. They account for 1.2%–4.6% of all intracranial tumors and are the most common non‐neuroepithelial intracranial neoplasm of children [1]. Most adamantinomatous craniopharyngiomas are well‐differentiated tumors, regarded as CNS WHO grade 1, with common *CTNNB1* mutations. Malignant craniopharyngiomas are exceedingly rare [7, 8, 9, 10, 11, 12, 13, 14, 15, 16, 17, 18, 19, 20, 21, 22, 23, 24, 25, 26, 27, 28, 29, 30, 31, 32, 33, 34, 35, 36, 37] and may occur de novo or arise from a benign craniopharyngioma [4, 16]. Regardless of origin, malignant craniopharyngiomas have a dismal prognosis and unclear treatment standards. Little is known about their pathogenesis. In this study, we perform an institutional review of adamantinomatous craniopharyngioma cases, present a unique case of malignant craniopharyngioma, and summarize clinicopathological findings of malignant craniopharyngiomas in the literature in an effort to better understand the pathogenesis of malignant craniopharyngiomas.

## 2 MATERIALS AND METHODS

### 2.1 Institutional case collection

A natural language search was performed in our institution's CoPath system to identify all adamantinomatous craniopharyngioma cases over the past 35 years (1989–2024). The patient clinicopathological characteristics (age, sex, and follow‐up data) were retrieved from hospital records.

### 2.2 Literature review

A literature review was conducted within PUBMED and SCOPUS using the keywords “craniopharyngioma” AND (“malignancy” OR “transformation” OR “neoplasm”) with the last query completed in July 2024. Different variables, including age, sex, tumor size, treatment, histologic variant, and outcome, were collected from the articles.

### 2.3 Statistical analysis

The Kaplan–Meier method was used to estimate the probability of survival. Progression‐free survival (PFS) was defined as the time between initial diagnosis of malignant craniopharyngioma and radiographic recurrence or last follow‐up. Overall survival (OS) was defined as the time from initial diagnosis of malignant craniopharyngioma to death or last follow‐up. For each factor, the Log‐rank test was performed to compare the survival times among the groups. R packages “survival” and “survminer” and GraphPad prism version 9.5.1 (GraphPad Software, San Diego, CA, USA) were used to perform the analyses. A *p*‐value <0.05 was considered statistically significant.

### 2.4 Immunohistochemistry

BAP1 and p53 immunohistochemistry was performed on 4 μm formalin‐fixed paraffin‐embedded (FFPE) slides using the Ventana Roche BenchMark Ultra IHC/ISH system following standard protocols. Mouse monoclonal antibodies against BAP1 (clone: C‐4; Santa Cruz; antibody dilution: 1:100) and p53 (clone: DO‐7; Roche) were used.

### 2.5 Next‐generation sequencing (NGS)

Nucleic acid was extracted from 10 unstained FFPE sections using QiaCube AllPrep extraction methods (QIAGEN, Germantown, MD) to prepare genomic material (DNA and RNA) for sequencing via the Precision Oncology Profile 300 (POP300). This test, based on the TruSight Oncology 500 assay backbone (Illumina, San Diego, CA), analyzes 337 genes for somatic sequence variants, and subsets of genes for structural changes and copy number gains. Testing was performed on the Illumina NextSeq 550Dx instrument (Illumina, San Diego, CA) with analysis by Local Run Manager and GenomOncology Software (Cleveland, OH). Additional information about POP300 can be found at https://www.testmenu.com/nebraska/Tests/1224493.

## 3 RESULTS

### 3.1 Institutional cohort of craniopharyngiomas

**FIGURE 1:**
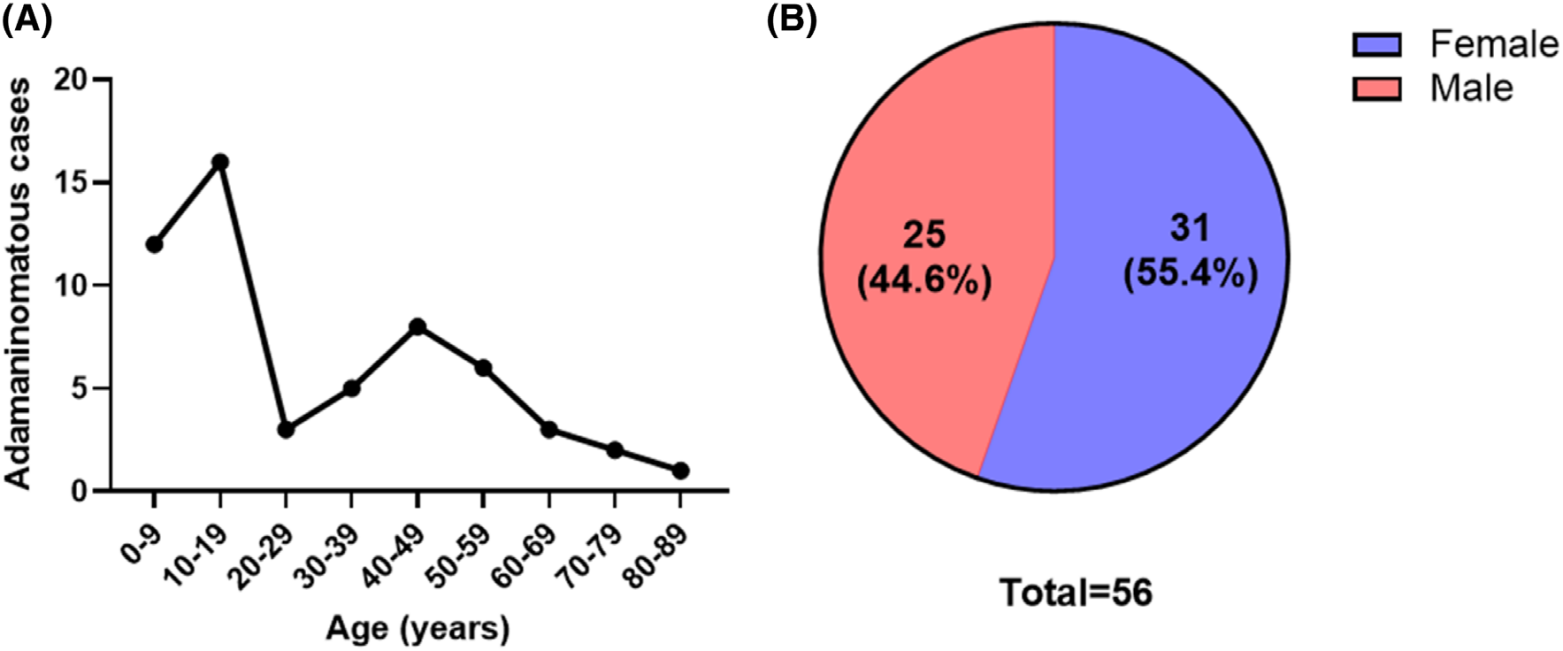
Our institutional cohort of 65 adamantinomatous craniopharyngiomas shows a bimodal age distribution (A) with a slight female predominance (B).

From 1989 to 2024, 65 adamantinomatous craniopharyngiomas were diagnosed in 56 patients at our institution. Age at initial diagnosis ranged from three to 80 years with a median age of 22 years and a bimodal distribution (peaks at 10–19 and 40–49 years; Figure 1A). There was a slight female predominance (31 females and 25 males; F/M ratio = 1.24; Figure 1B).

### 3.2 Malignant craniopharyngioma—Case presentation

Among the 65 cases from our institutional cohort, a unique case of malignant craniopharyngioma was identified in a 36‐year‐old male who was initially diagnosed with a benign adamantinomatous craniopharyngioma 16 years prior.

**FIGURE 2:**
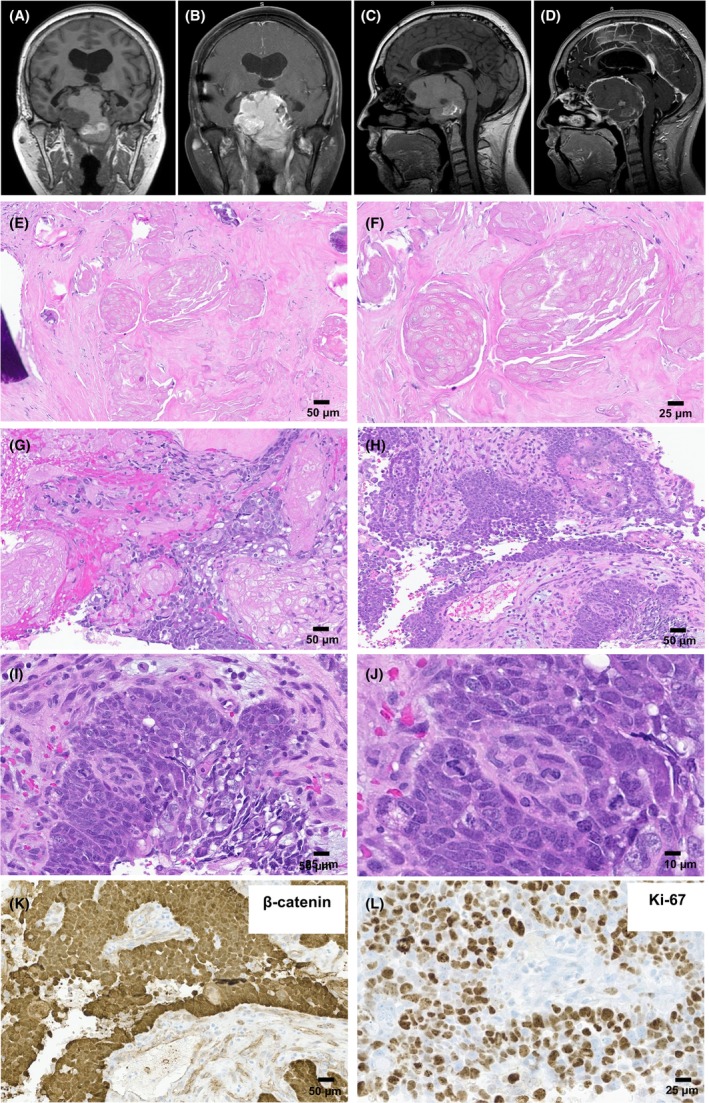
Radiological and pathological findings of the malignant craniopharyngioma case from our institution. Brain MRI demonstrated a 7.3 × 5.9 × 5.8 cm heterogeneous sellar mass with erosion of the adjacent central skull base structures, suprasellar extension, and mass effect on the adjacent brain parenchyma, optic chiasm, and circle of Willis arteries (A, axial T1; B, axial T1 post contrast; C, sagittal T1; D, sagittal T1 post contrast). H&E‐stained sections revealed a malignant neoplasm with retained adamantinomatous craniopharyngioma architecture (E–H) but significant nuclear pleomorphism and frequent mitotic figures (I and J). The malignant cells retained the aberrant nuclear reactivity for β‐catenin (K) and showed an elevated Ki‐67 labeling index, focally >50% (L).

He initially presented at age 20 with progressive weakness and was found to have a suprasellar cyst. Craniotomy and aspiration of the cyst were performed, and pathologic examination was consistent with adamantinomatous craniopharyngioma, CNS WHO grade 1. The slides from the initial craniotomy were not available for review. He received subsequent radiation therapy, ~50 Gy in 27 fractions, along with bleomycin via an Ommaya reservoir. Over the following 13 years, he underwent intermittent aspirations for cyst decompression and symptom control. At age 36, he presented with worsening headaches and nausea, and MRI revealed enlargement of the craniopharyngioma with areas of cystic change, calcification, and internal hemorrhage measuring 7.3 × 5.9 × 5.8 cm (Figure 2A–D). He was treated with intracavitary bleomycin and fluid removal with symptom improvement. Similar treatments and drainages were completed until late 2021 when brain imaging revealed significant enlargement of the tumor and acute intratumoral hemorrhage. A craniotomy with cyst wall fenestration and tumor biopsy was performed.

H&E sections from the biopsy specimen demonstrated areas of adamantinomatous craniopharyngioma with abundant wet keratin and dystrophic calcifications (Figure 2E,F). In some areas, the epithelial cells demonstrated pleomorphism with large nuclei, prominent nucleoli, high N:C ratios, frequent mitotic figures, and karyorrhectic debris (Figure 2G–J). Immunohistochemistry revealed the malignant epithelial cells to be strongly and diffusely positive for p40 with patchy positivity for CAM 5.2. Rare cells were positive for p53, and the Ki‐67 proliferation index was high, focally >50%. The tumor cells showed aberrant nuclear positivity for β‐catenin and were negative for BRAF p.V600E mutant protein expression. Taken together, the overall findings were consistent with a squamous cell carcinoma arising in the original adamantinomatous craniopharyngioma (Figure 2K,L).

Subsequently, the patient was treated with carboplatin and etoposide. Two months after the second surgery, he was placed on hospice and unfortunately passed away shortly thereafter.

### 3.3 Literature review

A comprehensive literature review identified 44 cases (current case included) of malignant craniopharyngioma. Most prior publications represent single case reports. Several small case series have been recently published [8, 28, 32, 38], the largest containing 7 cases [38]. A summary of the prior publications is noted in Table S1.

#### 3.3.1 Clinical features

**FIGURE 3:**
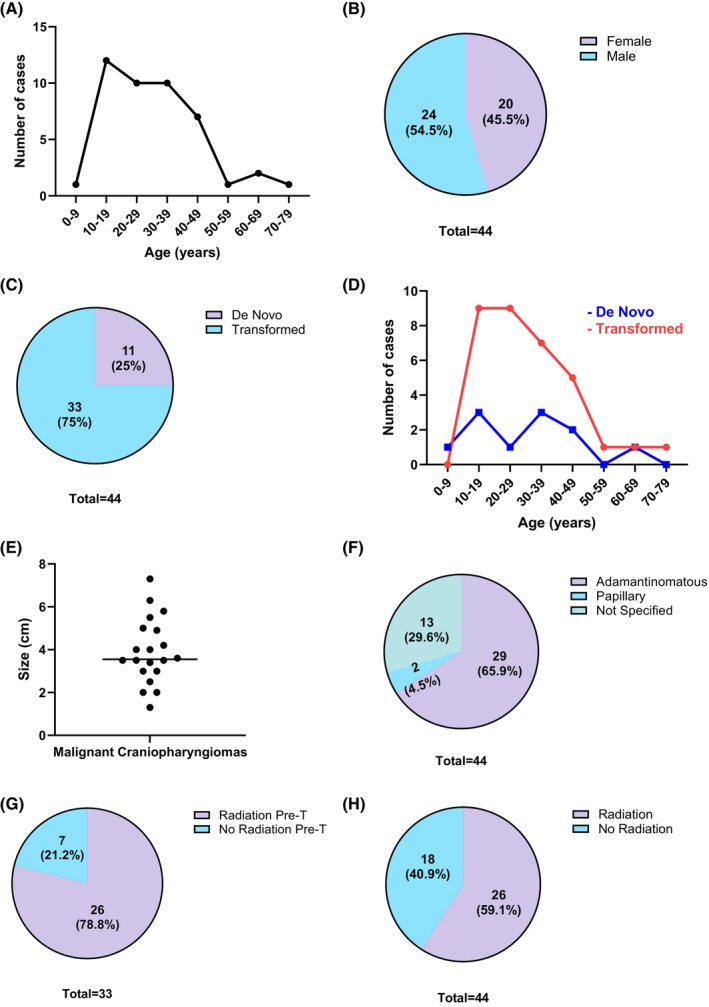
Characteristics of the 44 malignant craniopharyngiomas identified from the literature review (current case included). Tumors predominantly occurred in children and young adults (A), with a slight male predominance (B). Most tumors developed through malignant transformation (C). The age distribution of the 11 de novo cases showed a slightly bimodal distribution compared with the cases arising through malignant transformation (D). Malignant craniopharyngioma usually presented as a large sellar/suprasellar mass with a median size of 3.55 cm (E). Most tumors were of the adamantinomatous type (F). Although most patients received radiation before malignant transformation (G), 18 (40%) patients did not (H).

Malignant craniopharyngiomas occurred in patients of a wide age range but predominantly in young adults (median age 28 years; range 2.5–70 years old; Figure 3A). There was a slight male predominance (24 males and 20 females; M/F = 1.2; Figure 3B). Of the 44 cases, 11 (25%) arose de novo (Figure 3C) with a median age of 31 years (range 2.6–70 years; Figure 3D). There was also a slightly bimodal distribution (peaks at 10–19 and 30–49 years) similar to that of adamantinomatous craniopharyngiomas (Figure 1A). Among the 33 (75%) cases with malignant transformation, the median age at initial diagnosis of benign craniopharyngioma was 28 years (range 10–70 years) and the median interval to malignant transformation was 10 years (range 2–49 years; Figure 3D). The most common presenting symptoms were pan‐hypopituitarism (34%) and diabetes insipidus (18%).

#### 3.3.2 Imaging findings

Malignant craniopharyngiomas usually presented as a large, solid or cystic sellar/suprasellar mass. Reported tumor size ranged from 1.3 to 7.3 cm in greatest dimension with a median of 3.55 cm (Figure 3E). Three (6.8%) of the 44 cases showed invasion into the frontal lobe/cerebrum or adjacent sinuses. All 44 tumors had contrast enhancement, and 10 (22.7%) cases had calcifications.

#### 3.3.3 Histologic features

Malignant craniopharyngiomas, either de novo or secondary, were predominantly of the adamantinomatous type (29/44; 65.9%). Two were noted as the papillary type (4.5%), and the histology of the remaining 13 cases was not specified (Figure 3F). Of the 23 cases where histology of the malignant areas was described, the majority (18 cases; 78.3%) resembled squamous cell carcinoma. Additional histological types included sarcoma [20], small cell carcinoma [35], odontogenic ghost cell carcinoma [28], ameloblastic carcinoma [17], and myoepithelial carcinoma [28] (one case; 2.3% each).

#### 3.3.4 Therapeutic interventions

Of the 44 cases of malignant craniopharyngioma, most patients (32; 72.7%) received subtotal resection. Five (11.4%) patients received near‐total, and only two (4.5%) patients received gross‐total resection. Thirty (68.2%) patients received radiation either before or after transformation, and only 13 (29.5%) patients received chemotherapy.

Of the 44 malignant craniopharyngiomas, 33 (75%) were transformed from a previously benign craniopharyngioma (Figure 3C). Among these, 26 (78.8%) patients received radiation before the malignant transformation with doses ranging from 28 to 65.5 Gy in variable fractions. Seven (21.2%) patients did not receive any radiation prior to malignant transformation (Figure 3G). Together with the 11 malignant craniopharyngiomas that arose de novo, 18 (41%) malignant craniopharyngiomas occurred in patients without a history of prior radiation (Figure 3H).

#### 3.3.5 Outcomes

**FIGURE 4:**
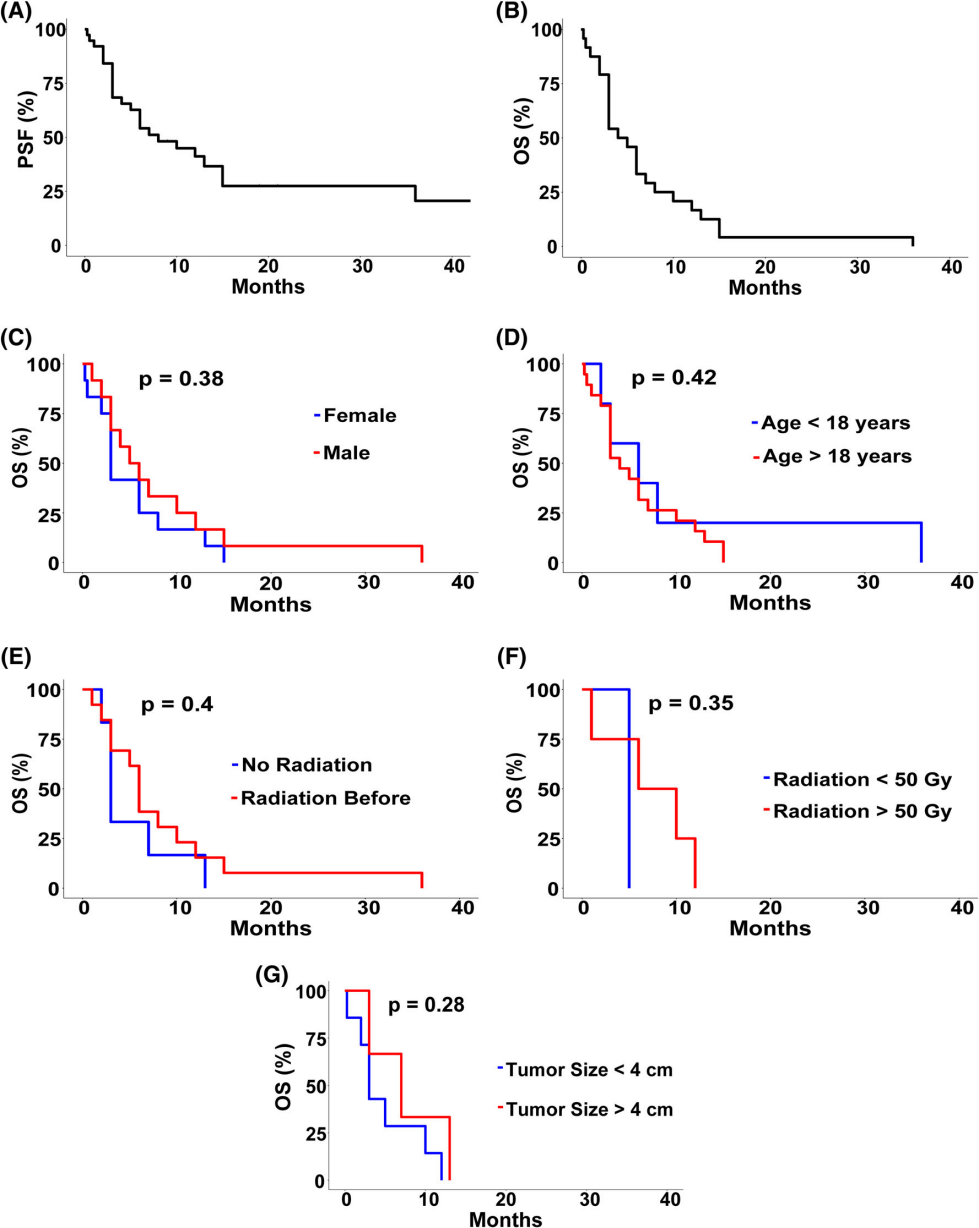
Outcomes of the 44 malignant craniopharyngiomas identified from the literature review (current case included). Median PFS and OS were nearly identical, with the median values being only 6 months (A and B), regardless of patient sex (C), age (D), history of radiation (E) and dosage (F), or tumor size (G).

Outcome data was available for 42 of the 44 cases of malignant craniopharyngiomas. Median PFS and OS were each 6 months (range: 1–13 years; Figure 4A,B). Patient sex, age, histologic variant, or tumor size did not seem to affect survival. Additionally, no significant survival difference was identified in patients who received radiation versus patients who did not (*p* > 0.5%; Figure 4C–G).

### 3.4 BAP1 and p53 immunohistochemistry and NGS results

**FIGURE 5:**
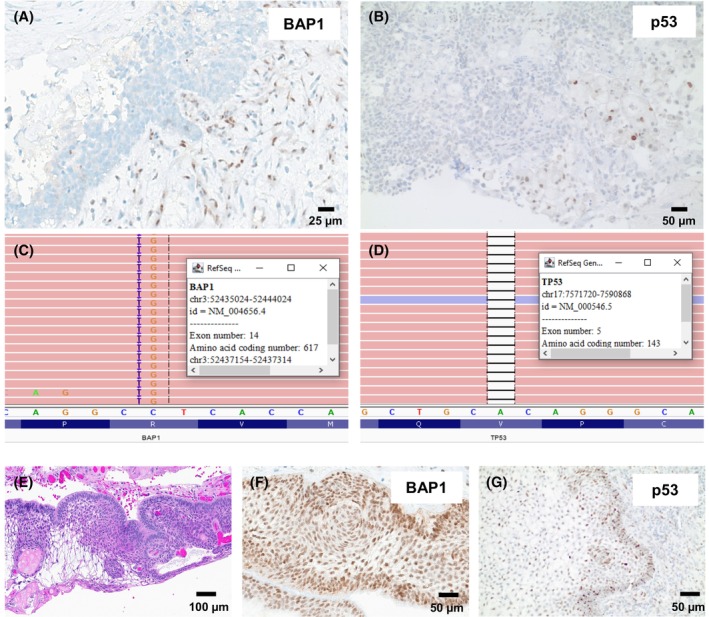
Described malignant craniopharyngioma case with complete loss of nuclear expression of BAP1 (A) and absent p53 expression (B). A representative benign adamantinomatous craniopharyngioma (E) showed retained nuclear expression for BAP1 (F) and scattered positivity for p53 (G), compatible with a wild‐type pattern. NGS revealed variants in BAP1 and TP53 (C and D) and CTNNB1 (not shown).

BAP1 and p53 immunohistochemistry was performed on the current malignant craniopharyngioma case. Malignant cells showed complete loss of nuclear BAP1 expression and were completely negative for p53 expression (Figure 5A,B). NGS (POP300) performed on the malignant craniopharyngioma confirmed a loss‐of‐function variant in BAP1 (c.1850delGinsCA;p.R617fs) with a variant allele frequency (VAF) of 17% (Figure 5C), a loss‐of‐function variant in TP53 (c.428delT;p.V143fs) with a VAF of 22% (Figure 5D), and a gain‐of‐function variant in CTNNB1 (c.110C>T;p.S37F) with a VAF of 20% (Table 1). No pathogenic or likely pathogenic variants were detected in *PBRM1*.

**TABLE 1:** Comparison of the sequencing results between two malignant craniopharyngiomas.

	Testing	*CTNNB1*	*TP53*	*BAP1*	*PBRM1*
Tamura et al.	FoundationOne CDx; Sanger sequencing	p.S33F	p.C135fs	p.L650fs	p.R1000*
Our patient	POP300	p.S37F	p.V143fs	p.R617fs	ND

Abbreviation: ND, not detected. * stop codon.

BAP1 and p53 immunohistochemistry was also performed on the 10 most recent cases of benign craniopharyngiomas from our institution, all of which showed intact nuclear expression for BAP1 and scattered positivity for p53, suggestive of a wild‐type pattern (Figure 5E–G).

## 4 DISCUSSION

Adamantinomatous craniopharyngiomas are generally considered benign tumors [1]. Malignant craniopharyngiomas, although exceedingly rare, do occur either de novo or via malignant transformation. Previously described histopathologic characteristics of malignant craniopharyngioma include high nuclear‐to‐cytoplasmic ratio, nuclear pleomorphism, hyperchromatic nuclei, increased mitotic activity, and coagulative necrosis [16]. Although squamous cell carcinoma is the most frequent malignant component, other rare histologic variants have also been reported, including sarcoma [20], small cell carcinoma [35], odontogenic ghost cell carcinoma [28], ameloblastic carcinoma [17], and myoepithelial carcinoma [28].

Regardless of histology, malignant craniopharyngioma seems to have a very dismal prognosis [17, 21, 31, 39]. Here, by an extensive literature review, we further demonstrated a poor prognosis of malignant craniopharyngiomas with median PFS and OS of only 6 months, independent of patient sex, age, tumor size, or radiation history. What makes it even more concerning is that malignant craniopharyngiomas tend to occur in children and young adults with a median age at diagnosis of only 28 years old. Therefore, we propose malignant craniopharyngioma be considered as a CNS WHO grade 4 malignancy, as some other authors have previously recommended [40].

To treat this high‐grade malignancy more effectively, a better understanding of its pathogenesis is essential. It has long been speculated that malignant craniopharyngiomas are associated with radiation in patients with a history of benign craniopharyngiomas [21, 23, 31, 38]; however, some authors have argued against this association [17, 31]. Our literature review revealed that although most (33; 75%) of the 44 malignant craniopharyngiomas developed in patients with a history of benign craniopharyngiomas, seven of these patients did not receive radiation therapy prior to malignant transformation. Together with the 11 (25%) malignant craniopharyngiomas that arose de novo, 18 (41%) malignant craniopharyngiomas occurred in patients without any prior radiation history. Therefore, factors apart from radiation may contribute to the carcinogenesis of malignant craniopharyngioma. Advanced molecular testing on more de novo and transformed malignant craniopharyngiomas is needed to explore these factors.

Due to its rarity, little is known about the molecular alterations in malignant craniopharyngiomas. In 2023, Tamura et al. shed some light on this topic [33]. By performing comprehensive genomic profiling (FoundationOne CDx) on a malignant craniopharyngioma, they identified several pathologic variants including BAP1 p.L650fs5, TP53 p.C135fs35, PBRM1 p.R1000*, and CTNNB1 p.S33F. Subsequent Sanger sequencing further highlighted BAP1, TP53, and PBRM1 variants in the invasive, malignant tumor but not in the benign, non‐transformed areas. They therefore proposed that the BAP1 and PBRM1 variants may be involved in the malignant transformation of adamantinomatous craniopharyngioma.

Here, we also tested BAP1 status in the current malignant craniopharyngioma case. By IHC, malignant tumor cells had loss of BAP1 nuclear expression. A large 337‐gene NGS panel (POP300) further confirmed loss‐of‐function variants in BAP1 and in TP53 and a gain‐of‐function hotspot variant in CTNNB1. The PBRM1 gene was also covered in the panel, with no variants detected. Mutations in CTNNB1 have a known association with adamantinomatous craniopharyngioma. Therefore, the presence of this CTNNB1 mutation further confirms our diagnosis of malignant craniopharyngioma.

The finding of BAP1 and TP53 variants in two cases of malignant craniopharyngioma (current case and case from Tamura et al.) suggests a potential role in malignant transformation of craniopharyngiomas. Both BAP1 frameshift variants (in codons 617 and 650) occur upstream of the nuclear localization signal (NLS) and would likely affect its function (as demonstrated by lack of BAP1 nuclear staining in our case; uniprot.org). *BAP1* is an established tumor suppressor gene involved in the regulation of processes including DNA damage repair, cell cycle control, chromatin modification, programmed cell death, and immune responses [41, 42]. Germline mutations of BAP1 are implicated in BAP1 tumor predisposition syndrome, and somatic mutations are tied to aggressive cancers including malignant mesothelioma, renal cell carcinoma, uveal melanoma, and others [43, 44, 45]. In both uveal melanoma and clear cell renal cell carcinoma, BAP1 mutations often occur after initial mutations of GNAQ/GNA11 oncogenic mutations or VHL mutations, respectively, and acquisition of the BAP1 mutation seems to link to poor prognosis and advanced disease states [46, 47].

It is unclear what caused the BAP1 mutations in these two malignant craniopharyngiomas. Both patients received treatment before malignant transformation. The patient reported by Tamura et al. underwent stereotactic radiotherapy (cyber knife, 27 Gy in 15 fractions). The current patient received radiation therapy (~50 Gy in 27 fractions) together with multiple rounds of bleomycin. Although it is reasonable to speculate that the BAP1 mutations might be induced by these treatments, currently there is no convincing evidence to support this hypothesis. More studies are required to fully understand the development of BAP1 mutations in malignant craniopharyngiomas.

In addition to a pathogenic variant in BAP1, we also identified a loss of function variant in TP53. TP53 alterations are not typical in benign craniopharyngiomas. Several earlier studies have documented increased p53 immunoreactivity in malignant craniopharyngiomas by immunohistochemistry [11, 20, 27]. However, it was not until 2023 when Tamura et al. demonstrated a TP53 variant in malignant craniopharyngioma [33] by sequencing for the first time. Both variants were identified in the TP53 DNA binding domain.

The role of BAP1 and TP53 mutations in the malignant transformation of craniopharyngiomas is currently unclear. One possibility is that these two mutations may act synergistically with the initial CTNNB1 mutation. CTNNB1 gain‐of‐function mutations are the most common genetic alterations in adamantinomatous craniopharyngiomas. They are believed to be driver mutations, resulting in unregulated Wnt/β‐catenin pathway signaling to promote increased cell proliferation and tumorigenesis [48]. Secondary inactivating mutations in the tumor suppressors BAP1 and/or TP53 may further drive malignant transformation by removing barriers to increased proliferation while allowing for defective DNA repair and unregulated cell cycle control [49, 50]. In addition, inactivating mutations in BAP1, a deubiquitinating enzyme, may hypothetically upregulate Wnt/β‐catenin signaling, as the key mechanism to shut down Wnt/β‐catenin signaling is through sequential phosphorylation in the N‐terminal region of β‐catenin followed by ubiquitin‐mediated proteolysis [51]. Of course, further research is needed to elucidate the precise role of BAP1 and TP53 mutations in the tumorigenesis of malignant craniopharyngiomas.

Identification of pathogenic BAP1 and TP53 variants in malignant craniopharyngioma may provide potential treatment targets. BAP1 has been a therapeutic target in other malignancies. For example, histone deacetylase inhibitors and EZH2 inhibitors have been developed targeting the role of BAP1 in chromatin modulation and transcriptional regulation, respectively [50, 52, 53, 54]. In addition, the use of PARP inhibitors and platinum chemotherapy agents has been suggested based on the role of BAP1 in DNA damage repair [55, 56]. Immunotherapies (anti‐PDL1 or anti‐CTLA4 therapies) have also been suggested for uveal melanomas due to the associations between BAP1 alterations and inflammatory tumor microenvironment, increased immune cell infiltration and increased immune checkpoint activation [57]. *TP53*, a tumor suppressor gene, is the most mutated gene in human cancers. While TP53 has generally been considered an undruggable target [58, 59], more recent investigations include the following: (1) restoration, stabilization, or limiting degradation of wild‐type p53 conformations, (2) inducing degradation or depletion of mutated p53 proteins, or (3) induced cell death specifically in cancer cells with deletions or mutations of the protein [60]. Small molecules, proteolysis targeting chimeras, immunotherapies, mRNA p53 vaccines, and gene therapy have also shown efficacy [59, 61, 62]. The emergence of these therapeutic options for BAP1‐ and TP53‐mutated cancers could then be of aid in malignant craniopharyngiomas.

Our study does have limitations. For example, our survival analyses did not reveal any significant differences in terms of patient sex, age, histologic variant, tumor size, or history of radiation. Only 44 cases were included in our analyses, despite an extensive literature search. The small sample size may have limited the statistical power of the analyses. Therefore, future studies with larger sample sizes are required to validate our results.

In conclusion, we report an institutional cohort of 65 adamantinomatous craniopharyngiomas with a classic bimodal age distribution (peaks at 10–19 and 40–49 years) and a unique case of malignant craniopharyngioma in a 36‐year‐old male who was initially diagnosed with a benign adamantinomatous craniopharyngioma 16 years prior. Our literature review demonstrated that malignant craniopharyngioma tended to occur in young adults with a dismal prognosis (median OS: 6 months) regardless of patient sex, age, histologic variant, tumor size, or history of radiation, though we acknowledge the small sample size due to the rarity of the tumor. Our malignant craniopharyngioma is the second case to show loss of function variants in BAP1 and TP53. BAP1 and TP53 may play an important role in the pathogenesis of malignant craniopharyngioma and may offer potential targets for therapeutic intervention. In addition, 18 (41%) malignant craniopharyngiomas occurred in patients without any history of radiation, suggesting that mechanisms other than radiation have contributed to their pathogenesis. Additional molecular testing on more malignant craniopharyngiomas is required to fully understand the carcinogenesis of this devastating malignancy.

## AUTHOR CONTRIBUTIONS

TA, IWZ, JMB, and MLC performed literature review. MLC, JLW, and NS provided clinical information. WZ performed the statistical analyses. JLC, SJC, and ACV analyzed the NGS results. TA and JC conceptualized the study and wrote the manuscript. All authors read and approved the final manuscript.

## FUNDING INFORMATION

This work was supported by interdepartmental fund to ACV and JC.

## CONFLICT OF INTEREST STATEMENT

The authors declare no conflicts of interest.

## ETHICS STATEMENT

This study was approved by the Institutional Review Board at the University of Nebraska Medical Center (IRB# 0518‐24) with a waiver of patient consent.

## Supporting information


**Table S1.** A summary of all the 44 malignant craniopharyngiomas published before July 2024 (current case included). F, female; GTR, gross‐total resection; NTR, near‐total resection; M, male; SCC, squamous cell carcinoma; STR, subtotal resection.

## Data Availability

The data that support the findings of this study are available from the corresponding author upon reasonable request.
